# Inflammatory patterns of antrochoanal polyps in the pediatric age group

**DOI:** 10.1186/s13223-019-0352-3

**Published:** 2019-06-20

**Authors:** Huiwen Zheng, Lixing Tang, Beibei Song, Xiaojian Yang, Ping Chu, Shujing Han, Pengpeng Wang, Jie Lu, Wentong Ge, Xin Ni

**Affiliations:** 1 Beijing Key Laboratory for Pediatric Diseases of Otolaryngology, Head and Neck Surgery, MOE Key Laboratory of Major Diseases in Children, Beijing Pediatric Research Institute, Beijing Children’s Hospital, Capital Medical University, National Center for Children’s Health, Beijing, China; 2 Head and Neck Surgery, Beijing Children’s Hospital, Capital Medical University, Beijing, China

**Keywords:** Antrochoanal polyps, Atopy, Cytokine

## Abstract

**Background:**

The pathogenesis and etiology of antrochoanal polyps (ACPs) remains obscure. This study aimed to characterize the inflammatory profiles and investigate the effect of atopy on the pathogenesis of pediatric ACPs.

**Methods:**

Thirty-three ACP patients and ten control subjects were enrolled from January to December 2017. The severity of individual nasal symptoms was scored on a visual analogue scale (VAS). The serum total immunoglobulin E (IgE) and cytokines level was measured by multiplexed luminex assay.

**Results:**

There was no significant difference in VAS scores and counts of inflammatory cells between atopic and nonatopic ACP. No difference in IFNγ, IL-4, IL-5, IL-13, IL-17A and IL-25 was found between control and whole ACP, nonatopic and atopic ACP. Significantly increased levels of IL-6 and IL-10 were found in ACP compared with control. For neutrophil chemotactic factor, significant increases of IL-8 and GRO were observed in ACP, but for eosinophil chemotactic factor, no difference was found in RANTES and GM-CSF. IL-6 level was positively correlated with IL-8, MCP1, and GRO level, and IL-10 level was positively correlated with IL-4 and IL-13 in ACP subjects.

**Conclusion:**

Nasal obstruction was the most common symptom in ACPs in children. Allergic condition may have a poor role in the pathogenesis of ACPs. IL-6 plays a crucial role in the pathogenesis of neutrophilic inflammation in patients with ACPs and may provide a new treatment strategy for ACPs in children. Treg cell associated cytokine IL-10 was involved in the inflammatory pathophysiological process of ACPs and played a certain regulatory role.

## Background

Antrochoanal polyps (ACPs) are benign polypoid lesions originating from the mucosa of the maxillary sinus, passing through the maxillary sinus ostium, and extending into the choana [1–3]. Although there are a few cases of bilateral ACPs demonstrated in literature, ACPs are invariably unilateral, and occur more commonly in children than in adults, with approximately 4–6% of all nasal polyps in general population and about 35% of all pediatric cases of nasal polyps [4, 5].

The pathogenesis and etiology of ACPs remains obscure, and cystic fibrosis and chronic sinusitis implicated [6]. Although some have proposed ACPs as a complication of chronic inflammatory antral disease, allergic polyps (abundant eosinophils) were found to be more common than inflammatory polyps (abundant neutrophils) among children by histopathology, and Chen and his colleagues reported that 50% of the patient had allergic diatheses in their review of ACPs in the pediatric population [7, 8].

Studies of comprehensive atopic response and inflammatory pattern have been conducted to understand the pathogenesis of nasal polyps and guide clinical therapies [9, 10], but such studies in ACPs are lacking. In this study, we attempted to investigate the effect of atopy on the pathogenesis of ACPs and characterize the inflammatory profiles in the pediatric population.

## Patients and methods

### Study population

This study was approved by the Ethics Committee of Beijing Children’s Hospital, Capital Medical University and written informed consent was obtained from all participants. This study was performed in thirty-three ACP patients who underwent endoscopic surgery from January to December 2017. The serum total immunoglobulin E (IgE) level was measured to evaluate the atopic status of the patients, and > 333 IU/ml (800 ng/ml) was considered as atopic ACP. Subjects who had immunodeficiencies, bronchiectasis, diabetes mellitus, cystic fibrosis, asthma or upper airway infections were excluded from the study. Control subjects were inferior turbinate mucosa from those underwent posterior nostril closure with no history of inflammatory nasal complaints. The severity of individual nasal symptoms was scored on a visual analogue scale (VAS) of 0 to 10.

### Immunological measurement

Fresh obtained tissue samples were frozen in liquid nitrogen and stored at − 80 °C. As reported [11], 0.1 g of each frozen tissue was added to 1 ml of 0.9% NaCl solution, followed by homogenized on ice and centrifuged at 3000 rpm for 10 min at 4 °C, and the supernatants were collected and stored at − 80 °C for further cytokines analysis. All samples were analyzed for interleukin (IL)-17A, Interferon (IFN)γ, growth related oncogene (GRO), IL-8, IL-13, macrophage inflammatory protein (MIP)-1α, granulocyte-macrophage colony-stimulating factor (GM-CSF), monocyte chemotactic protein-1 (MCP-1), regulated on activation, normal T cell expressed and secreted (RANTES) and tumor necrosis factor (TNF)α using human cytokine/chemokine panel I, IL-25, IL-1β, IL-5, IL-6, IL-10, IL-4, IL-21 and IL-33 using human Th17 panel (MILLIPLEX MAP KIT).

### Statistics

All statistical analyses were performed using the SPSS version 16.0 software. Data was expressed as box-and-whisker plots and median interquartile range (IQR). The Kruskal–Wallis H test was used to assess the significance of intergroup variability using paired comparisons, and the Mann Whitney U 2-tailed test was used to assess significance for between-group comparisons. The Spearman test was used to determine correlations. *P* values of less than 0.05 were considered statistically significant.

## Results

### Clinical data

In total, 43 subjects were enrolled in this study, including 33 ACP patients, and 10 control subjects. The clinical data was summarized in Table 1. Both atopic and nonatopic ACP patients had higher VAS scores of nasal discharge and overall burden compared with those in control. Nevertheless, no significant difference was observed in VAS scores of three other individual major symptoms and in inflammatory cells between atopic and nonatopic ACP.

**Table 1:** Patients’ clinical data

	Number				*P* value			
	Control (n = 10)	ACP (n = 33)	N-ACP (n = 23)	A-ACP (n = 10)	Control vs ACP	Control vs N-ACP	Control vs A-ACP	N-ACP vs A-ACP
Gender, male, n (%)	7 (70)	24 (72.7)	16 (69.6)	6 (60)	–	–	–	–
Age (years)	9 (7, 10)	9 (8, 11)	9 (7, 11)	9 (9, 11)	0.283	0.920	0.333	0.239
Serum IgE	–	558.75 (333.50, 899.75)	368 (311.88, 623.63)	1059 (902.88, 1726.38)	–	–	–	–
VAS score								
Sneeze	0 (0, 0.5)	0 (0, 1)	0 (0, 1)	0 (0, 1.25)	0.577	0.672	0.480	0.596
Nasal discharge	1 (0, 1)	2 (0, 2)	2 (0.25, 2.75)	2 (1.75, 3)	0.002	0.003	0.002	0.148
Nasal obstruction	2 (0.25, 2.75)	2 (2, 2)	2 (2, 2)	2 (1.75, 2.25)	0.262	0.423	0.306	0.628
Nasal itch	2 (2, 2)	0 (0, 1)	0 (0, 1)	0 (0, 0.5)	0.312	0.136	0.769	0.573
Overall burden	2 (2, 3)	4 (3, 5)	4 (3, 5)	5 (3, 7)	< 0.001	< 0.001	0.020	0.401
Counts of inflammatory cells in peripheral blood								
Neutrophile (1 × 10^9^/L)	4.12 (3.18, 4.85)	3.61 (2.88, 5.53)	3.37 (2.73, 4.08)	5.65 (3.27, 6.34)	0.878	0.358	0.153	0.056
Lymphocyte (1 × 10^9^/L)	3.16 (2.24, 4.27)	2.47 (2.02, 2.97)	2.56 (2.10, 3.18)	2.09 (1.99, 2.91)	0.140	0.214	0.082	0.254
Monocyte (1 × 10^9^/L)	0.39 (0.25, 0.46)	0.36 (0.28, 0.43)	0.33 (0.28, 0.42)	0.36 (0.28, 0.53)	0.887	0.562	0.818	0.584
Eosinophil (1 × 10^9^/L)	0.14 (0.07, 0.24)	0.10 (0.06, 0.16)	0.10 (0.08, 0.16)	0.10 (0.03, 0.21)	0.354	0.448	0.759	0.719

*N-ACP* nonatopic ACP, *A-ACP* atopic ACP

### Type 1, 2, and 17 cytokine levels in ACP

**Fig. 1:**
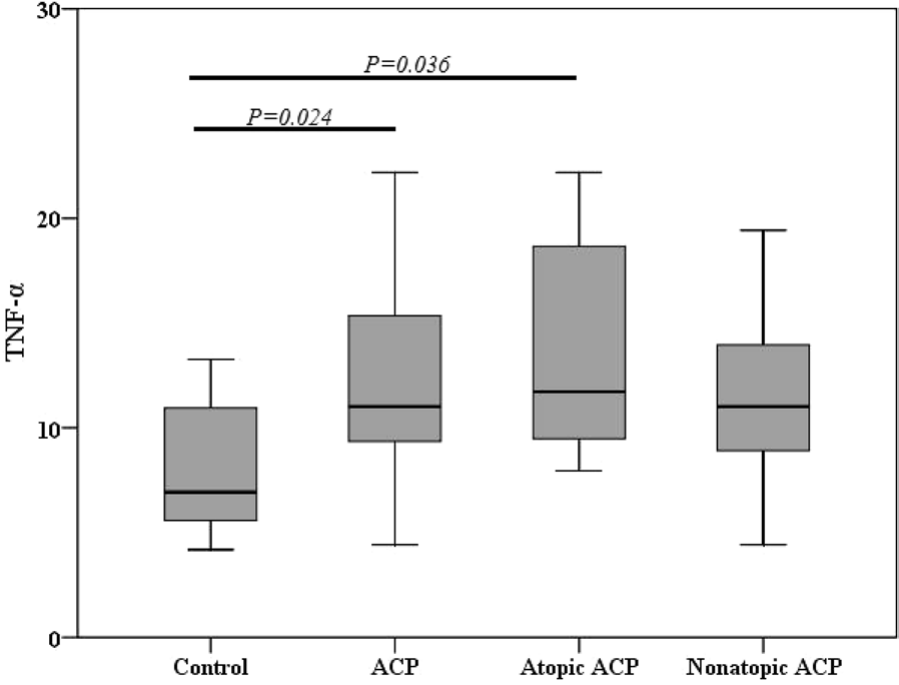
Levels of TNF-αin different groups. *ACP* antrochoanal polyp, *TNF-α* tumor necrosis factor-α

When compared all ACP and atopic ACP to control group, all had higher TNFα levels (control vs all ACP vs atopic ACP: 6.93 [5.23, 11.53] vs 11.01 [9.34, 15.61] vs 11.01 [8.71, 14.65]) (*P *= 0.024, and *P *= 0.036, respectively), but no difference was discovered between nonatopic ACP (11.73 [9.41, 20.07]) and control (Fig. 1). Besides, no significant difference in T_H_1 associated cytokine IFNγ was found among different study groups (control vs all ACP vs nonatopic ACP vs atopic ACP: 13.83 [8.62, 24.80] vs 20.18 [15.43, 30.16] vs 20.46 [16.09, 26.87] vs 19.72 [11.09, 56.82]).

No difference in the levels of T_H_2 related cytokines (IL-4, IL-5, and IL-13) was revealed among different study groups (control vs all ACP vs nonatopic ACP vs atopic ACP: 0.10 [0.04, 0.29] vs 0.06 [0.04, 0.09] vs 0.07 [0.05, 0.09] vs 0.06 [0.03, 0.19] for IL-4; 3.05 [1.90, 6.58] vs 4.14 [3.17, 5.84] vs 4.03 [3.36, 5.71] vs 5.58 [2.95, 9.23] for IL-5; 11.18 [6.22, 14.20] vs 12.52 [8.51, 15.96] vs 12.79 [9.58, 15.43] vs 14.05 [8.20, 18.21] for IL-13).

For T_H_17 cytokines (IL-17A and IL-25), no significant difference was observed between control and whole ACP, nonatopic and atopic ACP (control vs all ACP vs nonatopic ACP vs atopic ACP: 8.37 [5.17, 14.92] vs 8.32 [5.76, 9.64] vs 8.45 [5.99, 9.68] vs 6.23 [4.03, 19.43] for IL-17A, 0.00 [0.00, 0.05] vs 0.00 [0.00, 0.00] vs 0.00 [0.00, 0.00] vs 0.00 [0.00, 0.00] for IL-25).

### IL-6 and IL-10 expression in ACP

**Fig. 2:**
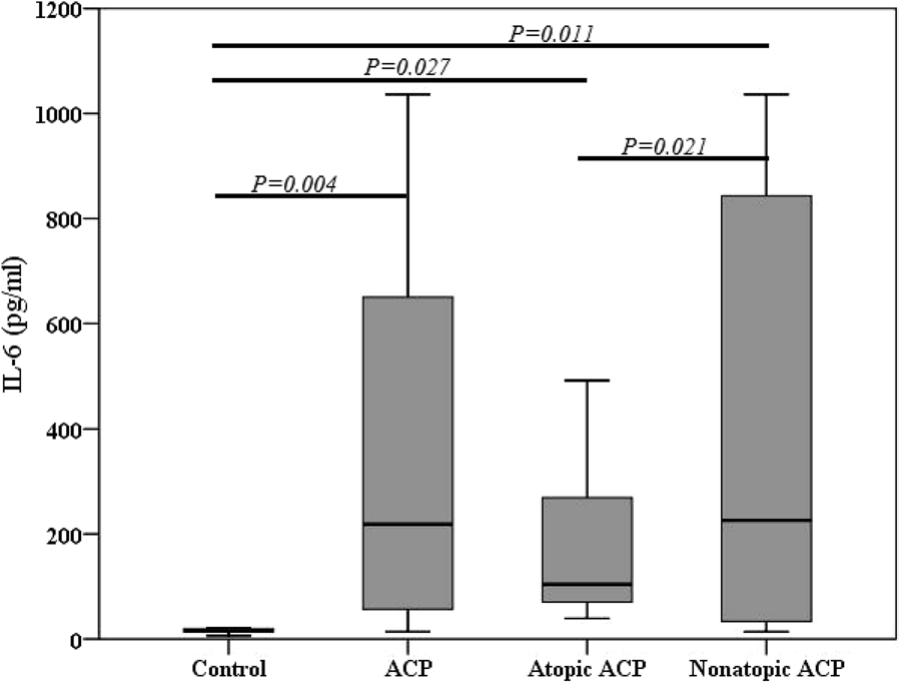
Levels of IL-6 in different groups. *ACP* antrochoanal polyp, *IL-6* interleukin-6

Significantly increased levels of IL-6, a proinflammatory cytokine, were found in whole ACP (218.44 [48.01, 717.18]), atopic (103.89 [68.88, 294.51]) and nonatopic ACP (273.84 [33.14, 2728.11]) compared with those in control (17.31 [11.69, 28.63]) (*P *= 0.004, *P *= 0.027, and *P *= 0.011, respectively), and between atopic and nonatopic ACP (*P *= 0.021) (Fig. 2).

**Fig. 3:**
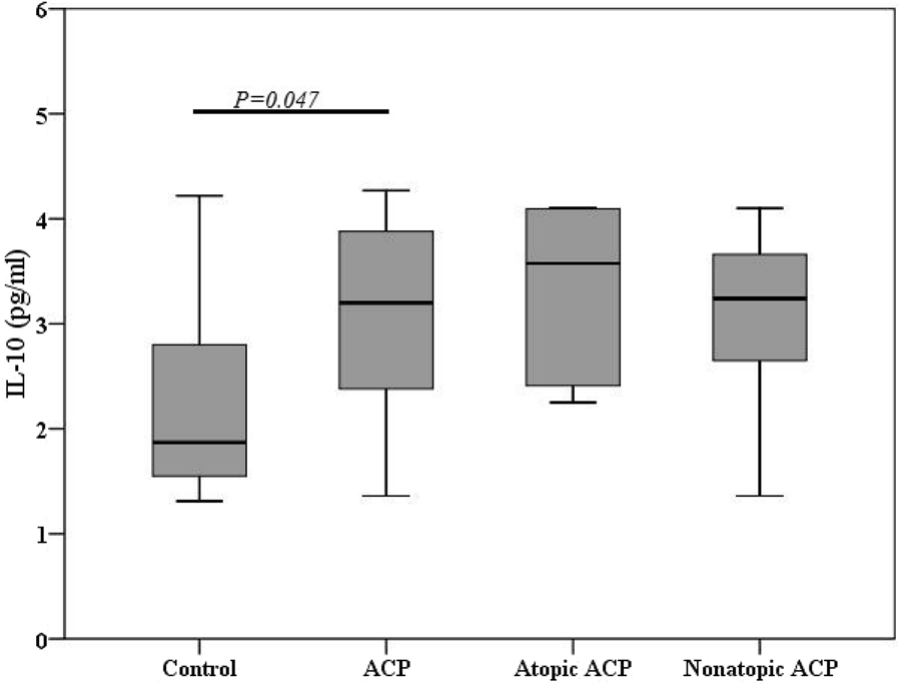
Levels of IL-10 in different groups. *ACP* Antrochoanal polyp, *IL-10* interleukin-10

Likewise, IL-10 (Fig. 3), a Treg related cytokine, was increased in whole ACP (3.20 [2.35, 3.90]) compared to controls (1.87 [1.49, 3.16]) (*P *= 0.047), but no significant difference was found in control compared with those in atopic (3.58 [2.35, 4.10]) and nonatopic ACP (3.24 [2.63, 3.67]).

### Chemokine expression in ACP

**Fig. 4:**
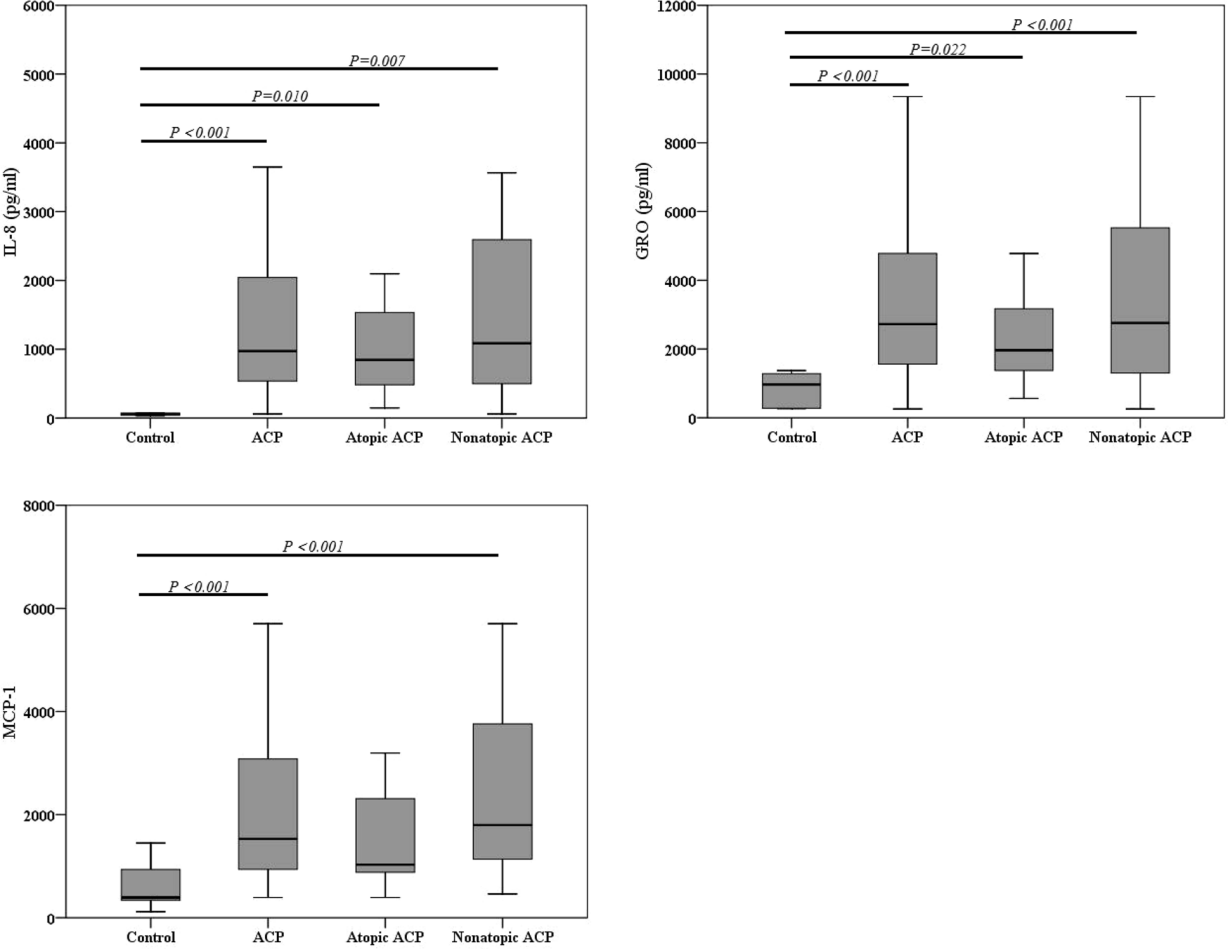
Levels of IL-8, GRO, and MCP-1 in different groups. *ACP* antrochoanal polyp, *IL-8* interleukin-8, *GRO* growth related oncogene, *MCP-1* monocyte chemotactic protein-1

IL-8, an important chemokine for neutrophils, was increased in whole ACP (971.93 [498.15, 2070.40]), atopic (844.10 [431.77, 1732.75]) and nonatopic ACP (1085.71 [478.35, 2706.34]) than that in control (69.82 [45.46, 205.68]) (*P* < 0.001, *P *= 0.010, and *P *= 0.007, respectively) (Fig. 4). Similar to IL-8, another neutrophil chemotactic factor GRO was observed significant increase in whole ACP (2724.81 [1511.21, 4980.43]), atopic (1965.91 [1333.36, 3218.19]) and nonatopic ACP (2754.90 [1106.60, 5525.88]) than that in control (968.74 [274.43, 1304.88]) (*P* < 0.001, *P *= 0.022, and *P* < 0.001, respectively) (Fig. 4).

MCP-1, which can attract different inflammatory cells, a statistically significant increase was found in whole ACP (1558.61 [939.01, 3147.30]) and nonatopic ACP (1796.90 [1103.00, 3919.84]) compared to control (392.34 [285.09, 1064.47]), whereas levels in atopic ACP did not differ from control group (Fig. 4).

No differences in RANTES and GM-CSF, chemoattractant for eosinophils, were found among different study groups (control vs all ACP vs nonatopic ACP vs atopic ACP: 3222.17 [1695.07, 6901.23] vs 3508.25 [1831.22, 6191.21] vs 3598.19 [2091.21, 5236.95] vs 2801.46 [1781.28, 8529.95] for RANTES; 4.15 [2.80, 8.59] vs 4.64 [3.82, 6.26] vs 4.45 [3.84, 5.91] vs 5.98 [3.53, 10.00] for GM–CSF).

MIP-1α ascribed to attract granulocytes was not differently expressed among different study groups (control vs all ACP vs nonatopic ACP vs atopic ACP: 16.84 [11.09, 46.06] vs 17.87 [14.63, 35.31] vs 18.03 [14.72, 33.77] vs 18.69 [14.70, 36.03]).

### The correlation between IL-6, IL-10 expression and inflammatory marker level

**Fig. 5:**
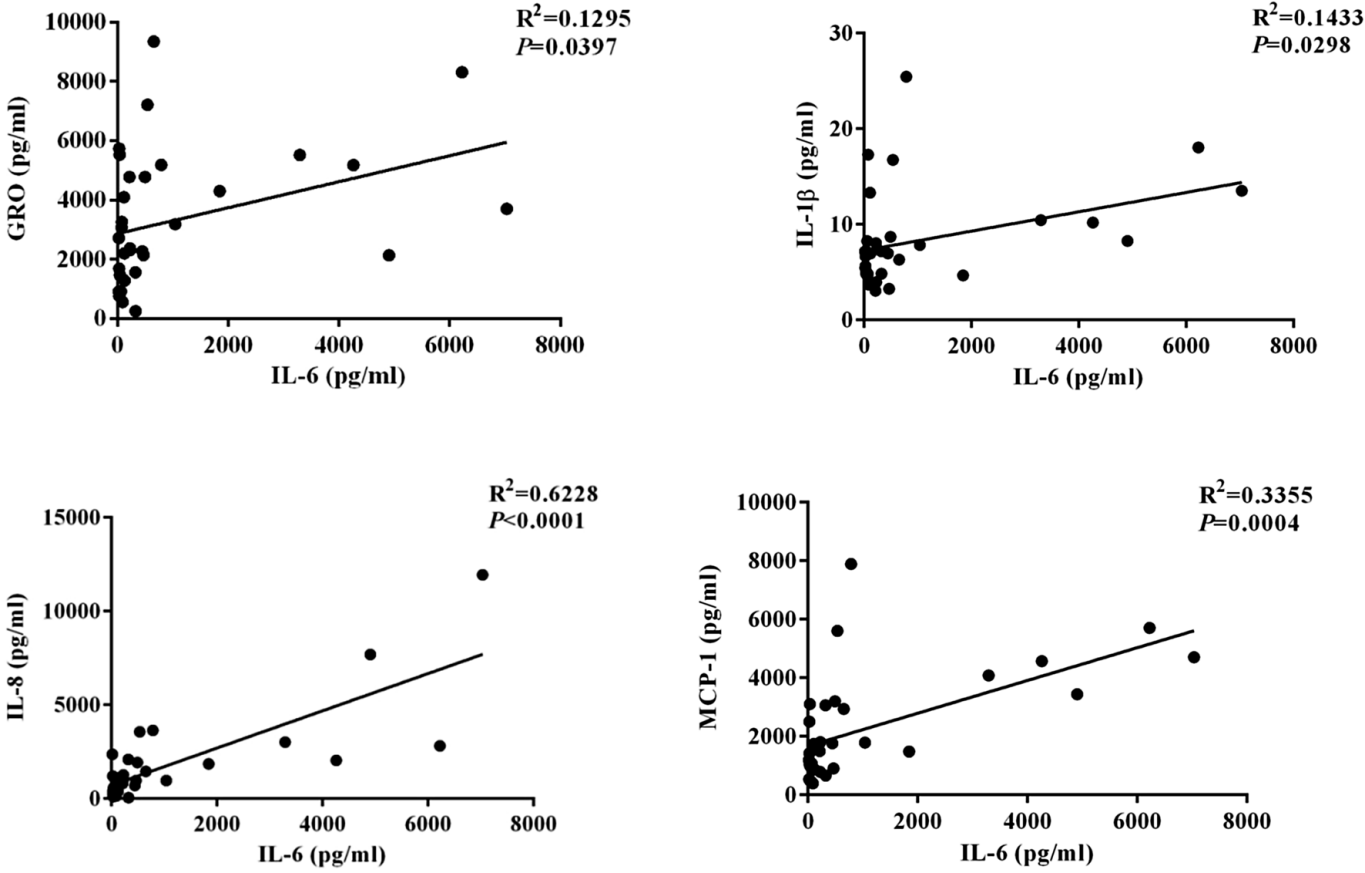
Correlation between IL-6 expression and GRO, IL-1β, IL-8, and MCP-1 level in antrochoanal polyps. *IL-6* interleukin-6, *GRO* growth related oncogene, *IL-1β* interleukin-β, *IL-8* interleukin-8, *MCP-1* monocyte chemotactic protein-1

**Fig. 6:**
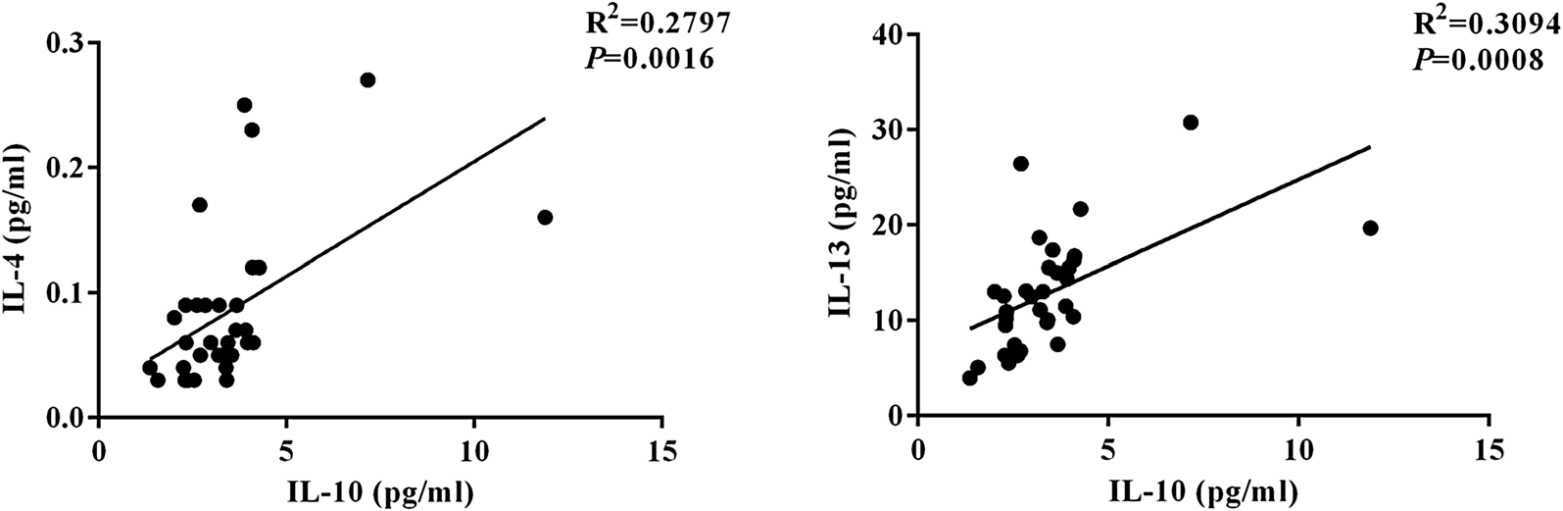
Correlation between IL-10 expression and IL-4 and IL-13 level in antrochoanal polyps. *IL-10* interleukin-10, *IL-4* interleukin-4, *IL-13* interleukin-13

IL-6 level was positively correlated with IL-8 level (*r*^*2*^ = 0.6228, *P* < 0.0001), IL-1β level (*r*^*2*^ = 0.1433, *P* = 0.0298), MCP1 level (*r*^*2*^ = 0.3355, *P* = 0.0004), and GRO level (*r*^*2*^ = 0.1295, *P* = 0.0397) in ACP subjects (Fig. 5). IL-10 level was positively correlated with IL-4 level (*r*^2^ = 0.2797, *P *= 0.0016), IL-13 level (*r*^2^ = 0.3094, *P* = 0.0008) in ACP subjects (Fig. 6).

## Discussion

ACPs in children are uncommon but occur at a higher rate than that in the adult population. Several previous studies showed that males were more common in ACPs [12–14], and our data confirmed this characteristic. Clinically, ACPs usually present with nasal obstruction and drainage, but the symptoms of dyspnea, epistaxis, dysphagia and postnasal drip were observed in severe cases [15]. In this study, the symptoms of ACPs were similar to those of many paranasal sinus diseases, including sneezing, rhinorrhea, itching, and nasal obstruction the most common symptom.

Allergic background of ACP patients was reported in some cases, which were significantly more common than inflammatory polyps among children on histopathology [8]. Previous studies have demonstrated that compared to nonatopic CRS, higher sneezing scores and reduced productivity and concentration was observed in atopic CRS patients [16]. But no studies focused on atopic and nonatopic ACPs patients, in the present study, we have identified 10 (30.3%, 10/33) allergic ACP cases, but failed to find a significant effect of atopy on symptomatic in ACP patients, which seems that the allergic condition may be coincident with the polyps but have no significantly contribution to their pathogenesis.

Up to now, the pathophysiology of ACPs has not yet been fully understood. Emerging evidence identified various cytokines and chemokines involved in the interaction of host and environmental factors during NP polypogenesis [17, 18], and comprehensive inflammatory pattern studies have been conducted to understand the pathogenesis of nasal polyps and guide clinical therapies [9, 10], but the key pathogenic mechanisms of ACPs are not completely elucidated. In this study, we investigated the characteristic of inflammatory profiles by Luminex in ACPs in the pediatric population to identify specific cytokines and to provide easily accessible biomarkers allocating patients specific therapies. To our knowledge, this is the first comprehensive study to investigate cytokines profiles for ACPs in children group.

Previous studies suggest that CRSwNP is usually characterized by type 2 T-helper (T_H_2) response and tissue eosinophilia with elevated level of IL-5 [18]. Though eosinophilic inflammation has been described in the nasal mucosa of NP patients, the eosinophil-related cytokine IL-5 level had no significant difference between ACPs and control. As for chemokines, RANTES known to attract eosinophils [19, 20] and M-CSF often appears in the context of recruitment, activation, and survival of eosinophils [20, 21], both of which were not upregulated in ACPs compared with control, further supporting the hypothesis that allergy could have a poor role in the pathogenesis of ACPs.

IL-6, a cytokine involved in the pathogenesis of various chronic inflammatory diseases, including crohn’s disease, rheumatoid arthritis, asthma, and lupus [22–24]. Previous studies indicated that elevated IL-6 protein was observed in polyp tissue compared to middle turbinate in the same patients with CRSwNP [25]. Consistent with CRSwNP, we found the level of IL-6 was significantly increased in whole ACP compared to control, which can be considered as a possible therapeutic target and studies with anti-IL-6 monoclonal antibodies show auspicious results. In addition, our study demonstrated that there was a positive correlation between IL-6 and proinflammatory cytokines IL-1β, meanwhile, the expression of neutrophil recruiting chemokines (GRO and IL-8) was positively correlated with IL-6, implying that IL-6 may enhance neutrophil recruitment to sites of infection.

Regulatory T (Treg) cells have been shown to inhibit Th1/Th2 response, and involve in the pathogenesis of respiratory allergic diseases [26]. Studies on regulatory T (Treg) cells in CRS are arising in recent years, but the results are controversial [27]. According to previous study, decreased Treg cells infiltration in ACP compared to NP and inferior turbinate mucosa from control on histopathology [28]. As a Treg related cytokine, IL-10 was increased in whole ACP compared to controls in our study. Besides, positively correlation between the expression of IL-10 and IL-4 and IL-13 was observed, indicating that Treg cell associated cytokine was involved in the inflammatory pathophysiological process of ACPs and played a certain regulatory role.

## Conclusion

In conclusion, we found that nasal obstruction was the most common symptom in ACPs in children. Allergic condition may have a poor role in the pathogenesis of ACPs. ACPs were characterized by elevated expression of IL-6, which was correlated with levels of various markers for neutrophil recruitment in ACPs tissues, suggesting that IL-6 plays a crucial role in the pathogenesis of neutrophilic inflammation in patients with ACPs and may provide a new treatment strategy for ACPs in children. Treg cell associated cytokine IL-10 was involved in the inflammatory pathophysiological process of ACPs and played a certain regulatory role.

## Data Availability

The datasets used and analysed during the current study are available from the corresponding author on reasonable request.

## Abbreviations

ACPs: antrochoanal polyps
VAS: visual analogue scale
IgE: immunoglobulin E
IL: interleukin
IFN: interferon
GRO: growth related oncogene
MIP: macrophage inflammatory protein
GM-CSF: granulocyte–macrophage colony-stimulating factor
MCP-1: monocyte chemotactic protein-1
RANTES: regulated on activation, normal T cell expressed and secreted
TNF: tumor necrosis factor
IQR: interquartile range
